# Large-scale reptile extinctions following European colonization of the Guadeloupe Islands

**DOI:** 10.1126/sciadv.abg2111

**Published:** 2021-05-19

**Authors:** Corentin Bochaton, Emmanuel Paradis, Salvador Bailon, Sandrine Grouard, Ivan Ineich, Arnaud Lenoble, Olivier Lorvelec, Anne Tresset, Nicole Boivin

**Affiliations:** 1 Max Planck Institute for the Science of Human History, Kahlaische Straße 10, D-07745 Jena, Germany.; 2 Laboratoire “Archéozoologie et Archéobotanique: Sociétés, Pratiques et Environnements" UMR 7209–CNRS, MNHN–Muséum national d’Histoire naturelle–Sorbonne Universités, 55 rue Buffon, CP 56, 75005 Paris, France.; 3 Institut de Systématique, Évolution, Biodiversité ISYEB–UMR 7205–CNRS, MNHN, UPMC, EPHE–Muséum national d’Histoire naturelle–Sorbonne Universités, 57 rue Cuvier, CP 30, 75005 Paris, France.; 4 PACEA–UMR CNRS 5199, Université de Bordeaux, 33 615 Pessac Cedex, France.; 5 Institut des Sciences de l’Évolution Montpellier ISEM, Université de Montpellier, IRD, CNRS, EPHE- Place Eugène Bataillon, CC 065 34095 Montpellier cedex 5, France.; 6 ESE, Ecology and Ecosystems Health, INRAE, Agrocampus Ouest, 65 rue de Saint-Brieuc, Bât. 15, CS 84215, 35042 Rennes, France.; 7 School of Social Science, University of Queensland, Brisbane, Queensland, Australia.; 8 Department of Anthropology, National Museum of Natural History, Smithsonian Institution, Washington, DC, USA.; 9 Department of Anthropology and Archaeology, University of Calgary, Calgary, Canada.

## Abstract

Large-scale extinction is one of the defining challenges of our time, as human processes fundamentally and irreversibly reshape global ecosystems. While the extinction of large animals with popular appeal garners widespread public and research interest, the importance of smaller, less “charismatic” species to ecosystem health is increasingly recognized. Benefitting from systematically collected fossil and archaeological archives, we examined snake and lizard extinctions in the Guadeloupe Islands of the Caribbean. Study of 43,000 bone remains across six islands revealed a massive extinction of 50 to 70% of Guadeloupe’s snakes and lizards following European colonization. In contrast, earlier Indigenous populations coexisted with snakes and lizards for thousands of years without affecting their diversity. Study of archaeological remains provides insights into the causes of snake and lizard extinctions and shows that failure to consider fossil-derived data probably contributes to substantial underestimation of human impacts to global biodiversity.

## Teaser

Indigenous human populations in Guadeloupe coexisted with island reptile species extinguished by later European colonialists.

## INTRODUCTION

The global biodiversity crisis is one of the most severe impacts of humans on Earth’s ecosystems (*1*). Assessments of present-day extinction rates by the International Union for Conservation of Nature indicate the disappearance of at least 1.07% of Earth’s vertebrate species over the past 500 years (*2*), a total that is probably grossly underestimated given the lack of subfossil data and poor documentation of taxa for most regions (*3*). Current estimates suggest, conservatively, an extinction rate increase in hundreds or even thousands of times over natural background extinction frequency (*3*, *4*). While reconstructing extinction patterns requires fossil and archaeological data, systematically studied regional archaeological and paleobiological datasets are few and far between. When historical data are present, they sometimes demonstrate that anthropogenic impacts extend very early, as seen in the case of the human-mediated impacts on megafauna beginning c. 50,000 years ago (*5*). However, except in a few cases, for example, the study of prehistoric bird extinctions in the Pacific (*6*), most available archaeological records of extinction are based on single sites or poor regional records. Furthermore, as in the field of ecology (*7*), the focus in archaeology and paleontology is very often on larger or charismatic faunal species, such as ungulates or large carnivores. Systematic study of neglected fauna such as reptiles and amphibians across multiple sites within a region has been a relatively limited undertaking to date.

Squamate reptile fossil remains offer an important opportunity to examine underexplored aspects of long-term anthropogenic impacts to biodiversity. Lizards and snakes likely arose between the Early Jurassic and Late Triassic and diversified worldwide, coming to occupy a wide range of ecological niches (*8*). As a function of the broader “taxonomic chauvinism” that has biased against the study of reptiles, amphibians, and invertebrates (*7*), the extinction rates (*9*) and ecological functions (*7*) of squamates have attracted less attention than those of other taxa. However, there is an increasing recognition that reptiles have an important role to play in ecosystem function, for example, in seed dispersal and pollination, nutrient transport, and as ecosystem engineers (*10*). Their loss, particularly from the tropics where much of their species diversity is concentrated (*11*), has implications for the maintenance of ecosystem services and broader biodiversity. While modern reptile extinctions are less well documented than other taxa (*12*), recent efforts to study anthropogenic impacts to these taxa suggest that at least one in five reptilian species is currently under threat (*10*). Understanding of the longer-term history of human impacts to reptilian fauna is strongly lacking.

The Caribbean region offers an exceptional opportunity to explore long-term human impacts on snakes and lizards. The Caribbean is a biodiversity hot spot in which present-day vertebrate faunas nonetheless represent only a remnant of richer species assemblages present in the past (*13*–*15*). While the causes of Caribbean extinctions have been debated for decades (*16*–*18*), recent studies point to Late Pleistocene climate change (*19*) and, particularly, human colonization and activities (*14*–*18*) as leading factors. Nonetheless, systematic archaeological and paleobiological research across the region has been patchy, and with the exception of a few studies of herpetofauna, research on terrestrial nonvolant biodiversity in the Caribbean has focused overwhelmingly on mammalian biodiversity (*13*, *14*, *17*, *18*, *20*). However, mammalian taxa represent only a very minor component of the native terrestrial nonflying fauna of the region (*14*). For example, in the Lesser Antilles, native nonvolant mammals are represented by only a single taxon on most islands. Instead, most of the native terrestrial nonvolant vertebrate fauna of the Lesser Antilles is composed of squamates. To date, however, snakes and lizards in the Caribbean have seen minimal paleontological or zooarchaeological investigation (*16*, *21*, *22*), in part, because suitable deposits preserving squamate remains are either rare (in the volcanic islands) and/or situated in highly anthropized coastal areas where they are extremely susceptible to disturbance or destruction (*23*).

The Guadeloupe Islands in the Lesser Antilles offer an important exception to this trend and, therefore, present a prime opportunity to study the role of climatic and anthropogenic factors in the extinction of snakes and lizards in the Caribbean. Not only do the Guadeloupe Islands host cave deposits containing both Late Pleistocene and Holocene fossiliferous layers but they have also seen a series of excavations of dozens of archaeological sites across the islands over the past 30 years. Critically, these excavations all involved systematic screening of archaeological sediments using 2- to 3-mm sieves, as well as retention of all faunal material, enabling recovery and study of the bones of small taxa. These consistent practices offer a rare and exceptional opportunity for the systematic study of squamate remains across multiple sites in one Caribbean island archipelago. Beyond this, the Guadeloupe Islands also provide the opportunity to look at several phases of human activity. These islands were occupied by Indigenous fisher-forager-horticulturalists mainly originating from South America, and later by colonial populations from Europe, Africa, and Asia, whose impact on biodiversity can be compared and discussed. The suitability of Guadeloupe as a case study for exploring shifts in squamate biodiversity also, of course, stems from the fact that islands are useful model systems, offering circumscribed spaces where past evolutionary, and anthropogenic, processes can be explored (*24*, *25*) and used to elaborate present-day biodiversity conservation strategies (*26*). The use of past data to set a baseline for human-induced extinction, predict future environmental impacts, and develop appropriate conservation strategies, particularly as part of the broader efforts of conservation paleobiology and applied zooarchaeology (*27*, *28*), is especially relevant in an insular context. Islands are some of the most imperiled ecosystems on the planet (*6*, *29*, *30*), their fragility offering clues as to what the future holds in more resilient continental ecosystems.

**Fig. 1:**
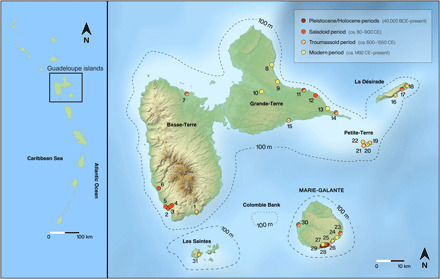
Map of the Guadeloupe Islands showing the location and phasing of the archaeological and paleontological assemblages investigated in the present study. Sites: 1: Pointe de Grande-Anse Trois Rivières; 2: 24, rue Schoelcher; 3: Place Saint-François; 4: Cathédrale de Basse-Terre; 5: Gare Maritime; 6: Embouchure de la Rivière Baillif; 7: Sainte-Rose La Ramée; 8: Grotte de l’Anse à l’Écu; 9: Grotte des Bambous; 10: Trou Lolo; 11: Morel; 12: Anse à l’Eau; 13: Grotte de l’Anse à la Gourde; 14: Anse à la Gourde; 15: Pointe du Helleux; 16: A l’Escalier; 17: Petite Rivière; 18: Pointe Gros Rempart 6; 19: Site du Phare; 20: Baleine Sud; 21: Caille à Bélasse; 22: Mouton de Bas; 23: Grotte du Morne Rita; 24: Grotte de la ravine Jean-François; 25: Tourlourous–Stade José Bade; 26: Grotte Blanchard; 27: Grotte Blanchard 2; 28: Abri Cadet 3; 29: Grotte Cadet 2; 30: Folle Anse; 31: Grande-Anse de Terre-de-Bas des Saintes. (Discontinuous lines correspond to the −100-m marine isobath)

To examine how humans have transformed the biodiversity of snakes and lizards in the Guadeloupe Islands from a deep-time perspective, we analyzed fossil squamate remains recovered from most of the known archaeological and paleontological deposits of the archipelago (Fig. 1 and table S1). Squamates represent not only the large majority but also, by far, the most diverse of the native vertebrate taxa of the Guadeloupe Islands, making them particularly suitable for studying transformations to the nonvolant vertebrate biodiversity of Guadeloupe through time. Through analysis of more than 43,000 individual squamate remains, we reconstructed the evolutionary history and diversity of these vertebrates over the past 40,000 years in relation to climate change and human-induced landscape modification.

### Regional setting

The Guadeloupe Islands occupy a central position in the Lesser Antillean insular chain linking South America to the Greater Antilles and Bahamas. The geological history of the Caribbean region is complex, with an outer volcanic arc formed of middle Cenozoic volcanic islands now covered with limestone and a younger inner arc of mountainous and still-active volcanic islands. The Guadeloupe archipelago extends across both arcs, yielding a set of islands of various ages and geologies. The largest and highest island, Basse-Terre (847 km^2^), is a mountainous volcanic island whose diverse topography and substantial geographic variability in annual rainfall have created a rich diversity of habitats (*31*). By contrast, the second largest island, Grande-Terre (586 km^2^), is a low and dry limestone island separated from Basse-Terre by a single marine channel less than 100 m in width. Together with two smaller limestone islands, La Désirade (21 km^2^) and Petite-Terre islets (1.49 km^2^), these two islands formed a single larger island during periods of low sea level, for example, during the Late Pleistocene (40,000 to 11,700 years ago). The two remaining islands are part of two different banks and were never connected to the others. Marie-Galante is a medium-sized (158 km^2^), low-elevation limestone island, while Les Saintes (~10 km^2^) is an assemblage of small volcanic islets that formed a single island of c. 200 km^2^ at the end of the Late Pleistocene (Fig. 1). The Guadeloupe Islands mostly emerged around 800,000 years ago, with the exception of La Désirade, which is much older at 2.7 million years (*32*).

Patterns of pre-Columbian human dispersal in the Caribbean region continue to be discussed and debated (*33*), meaning that the chronologies of diverse phases of colonization are not fully resolved, particularly south of the Guadeloupe passage. Still, limited archaeological and paleoecological (*34*) evidence supports colonization of at least some of the Guadeloupe islands by preceramic Indigenous groups during the Archaic period, perhaps as early as 5000 years before the present (B.P.). In the northern Lesser Antilles, Archaic sites are situated in coastal environments with good access to marine resources, and artifact assemblages include flint flakes together with some ground stone and shell (*Aliger gigas*) tools (*35*), supporting a foraging economy [intriguing starch evidence for crop cultivation from Archaic era sites in the southern Caribbean and northeast South America (*36*) is unfortunately open to critique on the grounds of potential contamination (*37*), nondiagnostic identifications, and a lack of systematic quantitative morphometrics]. Later, ceramic-making horticulturists known as the Saladoid culture settled the Lesser Antilles c. 2500 B.P., exploiting locally available marine and terrestrial resources that they supplemented with introduced plants and animals, including agouti (*Dasyprocta* sp.), armadillo (*Dasypus* sp.), guinea pig (*Cavia* sp.), deer (*Mazama*/*Odocoileus*), dog *(Canis lupus familiaris*), opossum (*Didelphis* sp.), peccary (*Tayassu* sp./*Pecari* sp.), and manioc (*Manihot esculenta*) (*15*, *38*). In Guadeloupe, Saladoid culture remains date back to perhaps 2000 B.P. (*39*), with the subsequent Troummassoid culture beginning c. 800/1000 CE (*40*), the transition between the two cultural periods perhaps associated with a phase of increased aridity (*41*). Columbus arrived in Guadeloupe in 1493, initiating a long period of European and Indigenous cohabitation before the start of French colonization in 1635 (*42*). Within 20 years, this colonization led to the disappearance of Guadeloupe’s Indigenous populations (*43*), the introduction of a number of novel and, in some cases, highly invasive species (*44*, *45*), and the progressive transformation of most of the islands for large-scale sugar cane cultivation and animal grazing (*15*, *46*). During the 17th century, agricultural exploitation was limited to the west coast of Basse-Terre and Marie-Galante, but in the 18th century, nearly all the primary forest of the archipelago was cleared with the exception of high-altitude forest (*31*).

The wild native nonvolant terrestrial fauna of the Guadeloupe Islands is currently dominated by reptiles, which display high rates of endemism, between 60 and 80% depending of the island (*45*). It is estimated that across the Lesser Antilles, densities of many reptiles and amphibians are several orders of magnitude higher than those of populations of birds and bats (*47*), and this is undoubtedly also true of Guadeloupe. Most Guadeloupe’s mammals are known to have been introduced during modern times (the three past centuries) and include commensals such as cats (*Felis catus*), rats (*Rattus rattus* and *Rattus norvegicus*), mongoose (*Herpestes auropunctatus*), and goats (*Capra hircus*) (*44*, *45*, *48*). In addition, archaeological evidence indicates that Indigenous human groups introduced the dog (*C. familiaris*) and the agouti (*Dasyprocta* sp.) to Guadeloupe (*48*). These two species still occur in the Guadeloupe archipelago today (*44*). While several Lesser Antillean islands are known to have been naturally colonized by nonvolant land mammals (*20*), evidence for these taxa in the Guadeloupe Islands before introduction by humans is lacking. Although pre-anthropic era faunal composition data are not available for most of the Guadeloupe islands, fossil data from Marie-Galante Island suggest that nonflying mammals were absent until the arrival of Indigenous populations (*49*). A recent ancient DNA (aDNA) study potentially supports a Pleistocene speciation/arrival of oryzomine rodents in Guadeloupe (*20*), but the small number of samples tested does not allow discussion of this hypothesis in regard to the fossil evidence. One or two species of now-extirpated terrestrial rodents (*Oryzomys* sp. and *Megalomys* sp.) have been described from archaeological deposits (*48*) and may have been present before Indigenous populations colonized Guadeloupe. The definitive identification of the bones of these two species remains to be undertaken, but one of them is likely to correspond to the *Antillomys rayi* identified through aDNA study (*20*).

## RESULTS

Fossil remains attributable to 16 different taxa were identified across the 31 sites and six islands sampled in the study (table S2). Fossil data were classified into four temporal phases: (i) Late Pleistocene (32,000 to 11 650 B.P.); (ii) pre-anthropic Holocene (11,650 to 2450 B.P.); (iii) Indigenous habitation period (combining the Saladoid and Troumassoid periods) (2450 to 458 B.P.); and (iv) modern, encompassing the period after 458 B.P. until the present. The dataset obtained was used to reconstruct changes in squamate diversity on the different islands and across the Guadeloupe archipelago as a whole.

### Transformation through time of squamate diversity on the individual islands of Guadeloupe

Variable patterns of squamate diversity and extinction/extirpation were observed across the diverse islands of Guadeloupe. While estimates of the minimum number of taxa on each island were correlated with island size (Pearson’s *r*; *R*^2^ = 0.68; *P* < 0.05), very little data were available for the small islets of Les Saintes and Petite-Terre, precluding evaluation of the significance of the diversity estimation for these islet clusters. Species richness estimators and rarefaction curves that are often used to correct biodiversity sampling effects (*50*) were not applicable as biodiversity sampling varies by site type and is influenced by accumulator behaviors.

#### Marie-Galante Island

**Fig. 2:**
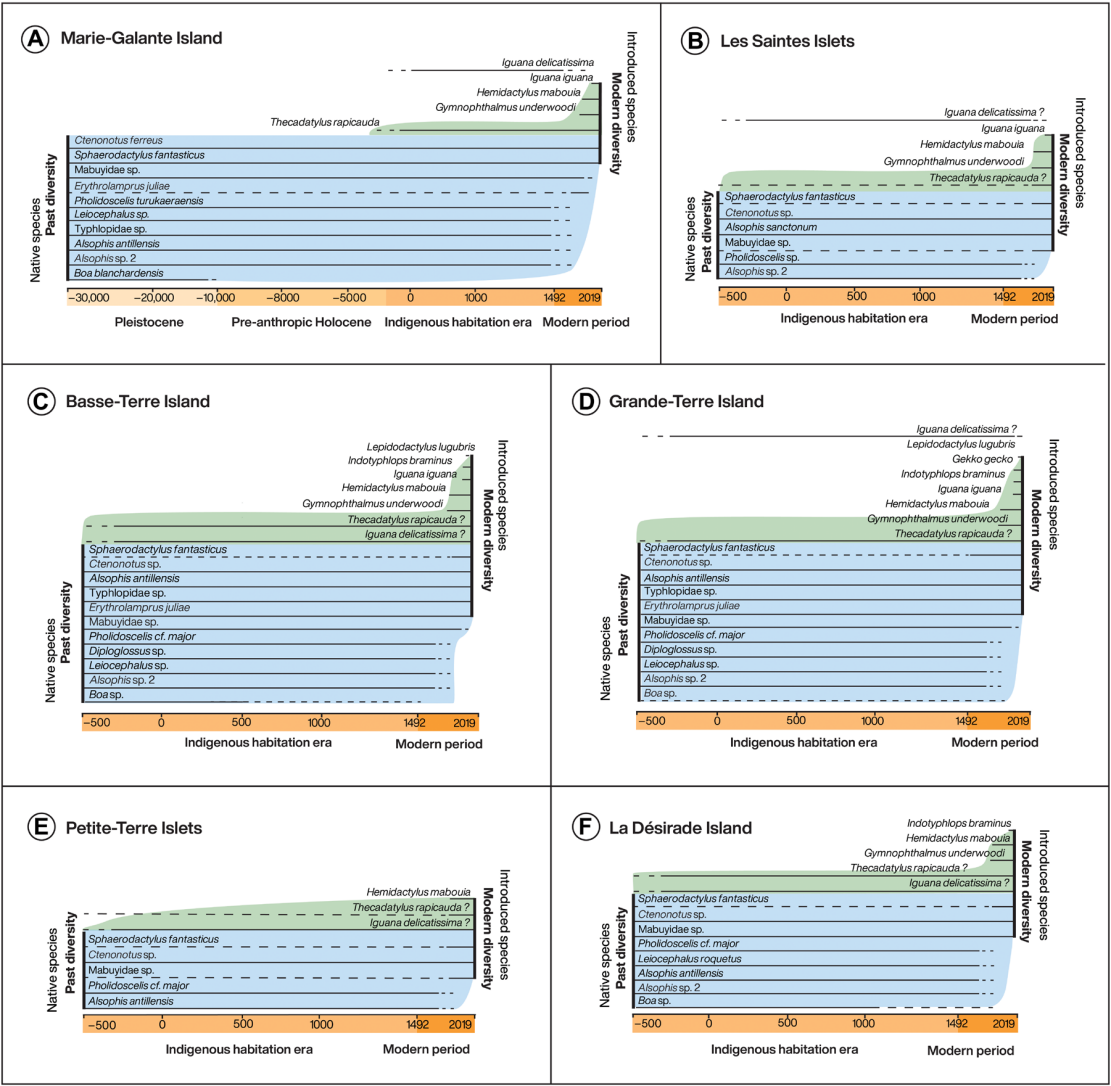
Transformation of squamate diversity through time on the islands and islet clusters of Guadeloupe, showing the progressive extinction of native taxa and their replacement by newly introduced species. Taxa for which occurrences are attested by fossil evidence are represented by plain lines and those for which past occurrence is extrapolated on the basis of modern data, possible recovery bias, and biogeographic hypotheses are indicated by dashed lines. Blue parts of the plots correspond to native taxa and green parts correspond to introduced ones. Question marks indicate the dubious introduced status of *I. delicatissima* and *T. rapicauda* that is suggested by fossil evidence only on Marie-Galante Island. For fossil data, the taxon level used (species, genus, or family) corresponds to the one provided by paleontological analysis. The start of the Indigenous habitation era for each island was arbitrarily fixed at 2450 B.P. (500 BCE), which is the average date for the onset of the Ceramic Age in the Caribbean. (**A**) Marie-Galante Island; (**B**) Les Saintes Islets; (**C**) Basse-Terre Island; (**D**) Grande-Terre Island; (**E**) Petite-Terre Islets; (**F**) La Désirade Island.

This is the only island for which both Pleistocene and Early Holocene fossil data were available (Fig. 2A). Five taxa of lizards and four of snakes, identifiable to genus or species level, were found in the Pleistocene layers of the three sites with evidence for this epoch (Blanchard cave, Cadet 2 cave, and Cadet 3 shelter deposits) (table S2). Only *Boa blanchardensis* was restricted to the Pleistocene layers. This snake also did not occur in pre-Columbian archaeological deposits at other sites on Marie-Galante (table S2). In pre-Columbian archaeological layers, two additional lizards, probably introduced during that period, were found: *Iguana delicatissima* and *Thecadactylus rapicauda*. A specimen of a now-extinct endemic snake, *Erythrolamprus juliae mariae* (*51*), was collected on the island in the 20th century, but this species, despite being endemic and thus very likely present on the island for a long period of time, has not, so far, been identified in the fossil record. The current squamate fauna of Marie-Galante is composed of six lizards, of which four were introduced in the modern era and only two were already present on the island in the period of Indigenous habitation. All other “native” taxa, representing 72% of the native reptiles, went extinct or were extirpated during modern times. Recently introduced species represent 50% of the current squamate diversity of Marie-Galante (table S4).

#### Les Saintes Islets

A single pre-Columbian archaeological bone assemblage was available on these islets. This single site revealed the past presence of three taxa of lizards and two of snakes in the islet cluster (Fig. 2B), but by combining modern data and comparing the assemblage to that on other islands in Guadeloupe, it was possible to postulate the past occurrence of three additional lizards. The very small lizard, *Sphaerodactylus*, is today represented by endemic species on each of the main Guadeloupe islands (*45*, *52*), clearly indicating the presence of this genus in Guadeloupe for a long period of time. For this reason, we suggest the past occurrence of these small geckos on all Guadeloupe islands even when they are, so far, absent from the fossil record. We also hypothesize the past occurrence of Mabuyidae on Les Saintes as this taxon was present on every other main Guadeloupe island, with at least six species endemic to the archipelago (*53*). By analogy with the archaeological record of other Guadeloupe islands, as well as its current presence on the island, we also postulate the past occurrence of *T. rapicauda* on Les Saintes. Data concerning the current diversity on Les Saintes Islets demonstrate that two lizards and one snake went extinct after the Indigenous habitation period, indicating a minimal extinction/extirpation percentage of 37% of pre-Columbian era fauna for Les Saintes. This rate is, however, probably underestimated because of the lack of fossil data since only one site could be investigated. If the fauna present before European arrival was in fact similar in diversity to that we observed on the other islands, then the percentage of species undergoing extinction or extirpation in the modern era would reach approximately 61% (table S4). These islets are currently inhabited by five taxa that were already present during the period of pre-Columbian habitation, representing 60% of the current squamate diversity of the area (table S4).

#### Basse-Terre Island

Seven sites were examined in total on Basse-Terre Island. Archaeological data indicate the pre-Columbian presence of, minimally, seven taxa of lizards and five of snakes on the island, including *Boa* sp (Fig. 2C). The latter was identified in the form of a manufactured bead, and we also demonstrate that it was present in the past on La Désirade, which is part of the same island bank as Basse-Terre Island (*54*). Unlike most squamate taxa occurring in the archaeological record, *Boa* snakes were never described by historical chroniclers in Guadeloupe, rendering their extinction date very uncertain. Modern data indicate that six of the taxa that were present on Basse-Terre Island in the Indigenous habitation period are today extinct and have been replaced by four newly introduced species. This indicates the extinction/extirpation of 46% of the squamates present during the Indigenous habitation era, with extant squamates representing 58% of the current squamate diversity of Basse-Terre (table S4).

#### Grande-Terre Island

The squamate diversity of Grande-Terre in the period of pre-Columbian habitation was similar to Basse-Terre (Fig. 2D). The only difference in zooarchaeological diversity between the two islands was the absence of *Boa* in Grande-Terre, but its past presence there can be hypothesized given that Basse-Terre and Grande-Terre formed a single island mass during the Pleistocene. Fifty-three percent of the native pre-Columbian taxa went extinct on Grande-Terre in modern times, and surviving native species constitute 50% of the current squamate diversity of the island, with the other 50% composed of introduced species (table S4).

#### Petite-Terre Islets

The little available data for the Petite-Terre islets revealed the presence of four taxa in the era of Indigenous habitation (Fig. 2E). Our hypothesis regarding past occurrences of taxa currently present on the islets follows the same arguments as for the Les Saintes islets. Four taxa is almost certainly an underestimation of the true past diversity given that these islets were connected to Basse-Terre and Grande-Terre during the Pleistocene. Data concerning present-day squamate diversity on these islets indicate the presence of six species, five that were already present in Guadeloupe during the period of pre-Columbian habitation and one that was probably recently introduced. Combining fossil and present-day data enabled the estimation of a minimal diversity of at least seven squamate species on Petite-Terre during the period of Indigenous habitation, but diversity could well have been as high as on Basse-Terre (13 taxa). Depending on the accepted degree of uncertainty, between 28 and 61% of squamates on the Petite-Terre islets are estimated to have gone extinct during modern times. Taxa present during pre-Columbian periods still represent 85% of the squamate diversity of this cluster, to which very few taxa have been recently added, likely as a result of human-mediated introductions.

#### La Désirade Island

Data collected on La Désirade demonstrate the occurrence of nine squamate taxa present during the period of Indigenous habitation, indicating a minimal past diversity on the island of 10 taxa (Fig. 2F). Only five of these taxa still occur on the island today, which represents an extinction/extirpation of 50% of the island’s squamates in the period following European colonization. However, given that La Désirade was connected to Basse-Terre and Grande-Terre during periods of low sea level (Fig. 1) (*32*), actual past squamate diversity on La Désirade may have been similar to that on these other islands, which could make this rate as high as 61% (table S4). The modern squamate diversity of La Désirade is composed of eight species, of which 60% were already present in pre-Columbian times and the remainder introduced by later human populations (table S3).

### Global trends

Fossil data enabling the estimation of Late Pleistocene and Early Holocene squamate diversity in Guadeloupe is unavailable for most islands, with the exception of Marie-Galante. Other lines of evidence nonetheless suggest that squamate diversity in Guadeloupe before the arrival of humans was similar to that in the period of Indigenous habitation. When modern and fossil data from the region are taken into account, it seems likely that the 13 taxa identified in pre-Columbian Basse-Terre reflect most of the full set of taxa that could potentially have been present in the past in this part of the Lesser Antilles. Furthermore, on Marie-Galante Island, Pleistocene and Early Holocene squamate diversity is very similar to that during the period of Indigenous habitation. Marie-Galante was only weakly affected by the Pleistocene/Holocene climatic transition, with only a single taxon (*B. blanchardensis*) appearing to have been extirpated at that time; otherwise, the island’s squamate diversity remained stable from the Pleistocene through to the period of Indigenous habitation. While this suggests that the climatic perturbations of the Late Pleistocene and Early Holocene had only a minor impact on squamate diversity in the Guadeloupe Islands, some uncertainty remains concerning the small islands of Les Saintes and Petite-Terre. As sea levels rose with postglacial warming, these islands saw substantial size reduction (see Fig. 1) and fragmented into small islets. It is likely that these transformations had an important impact on the faunal composition of the islands, leading to reductions already by the mid-Holocene, before human colonization. Unfortunately, available data are insufficient to explore this putative shift.

The pre-Columbian cultural era may have seen the introduction of two new taxa, *T. rapicauda* and *I. delicatissima*, to Guadeloupe. These taxa are absent in the pre-anthropic fossil assemblages from Marie-Galante Island but appear in the Indigenous habitation period and later assemblages on Marie-Galante as well as on all the other islands of the Guadeloupe archipelago. On the basis of what we know from modern populations, *T. rapicauda* is a Central and South American species. The *T. rapicauda* population on Guadeloupe may have not yet diverged from its continental counterpart (*45*), suggesting a more recent introduction that could fit with the hypothesis of an Indigenous translocation to the archipelago. With respect to *I. delicatissima*, aDNA analysis suggests that it probably dispersed in the Lesser Antilles during the past millennia and may have been transported there by Indigenous populations (*55*). If hypotheses about the putative origins of these species are correct, then the pre-Columbian peopling of the Guadeloupe Islands led to an increase in squamate diversity but apparently did not result in any extinction or extirpation of reptile taxa. During the period of Indigenous habitation, the squamate taxa on the different islands of Guadeloupe included 62 individual insular “populations,” either identified in the fossil record or where past occurrences could be extrapolated on the basis of the current or historical occurrence of endemic taxa. Postulating the past occurrence of the same taxa on all the islands that previously formed a single island at low sea level, this total could have been as high as 76 populations.

**Fig. 3:**
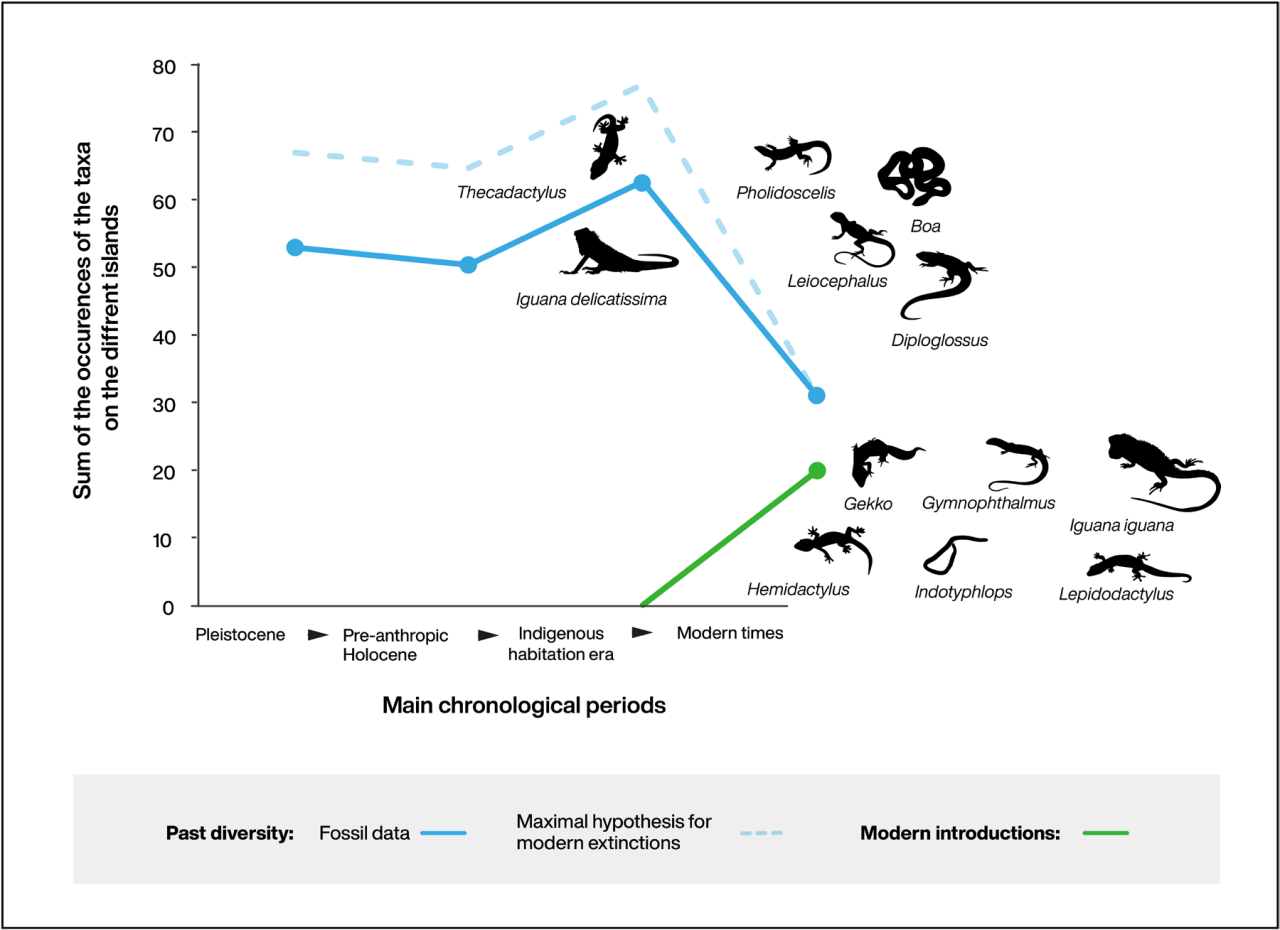
Change in the occurrences of taxa on the different Guadeloupe islands through time based on fossil data. An occurrence corresponds to the presence of a given taxon on any of the six islands or islet clusters. Fossil data for the Pleistocene and pre-anthropic Holocene phases are an extrapolation from the scenario observed on Marie-Galante Island.

Comparison with modern data from Guadeloupe suggests that 50 to 59% of squamate populations became extinct or were extirpated following the arrival of Columbus and subsequent European colonization of the Guadeloupe Islands in the 17th century. This includes the extinction or complete extirpation of four genera (*Pholidoscelis*, *Leiocephalus*, *Diploglossus*, and *Boa*) and at least eight species. These extirpations and extinctions coincided with a strong faunal turnover that began with the introduction to the Guadeloupe Islands of six new genera or species of lizard (*Gekko*, *Gymnophthalmus*, *Hemidactylus*, *Iguana iguana*, *Indotyphlops*, and *Lepidodactylus*) (Fig. 3). These newly introduced species represent 39% of present-day squamate-specific diversity in Guadeloupe.

### Modeling of extinction factors

**Fig. 4:**
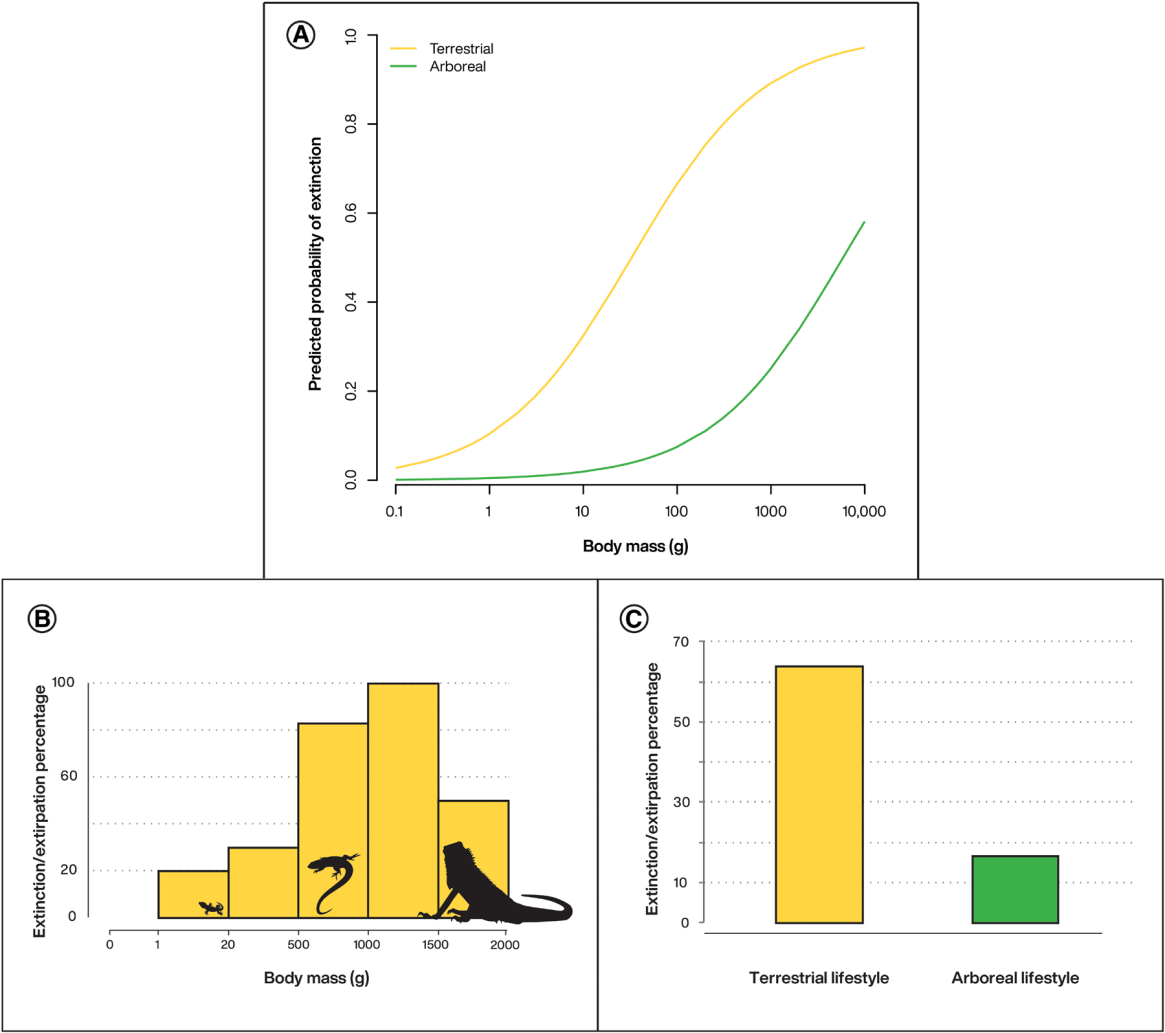
Relationship between a proportion of Guadeloupe squamates that experienced extinction/extirpation, body mass, and lifestyle. (**A**) Relationship between these variables following an OU model; distribution of the body mass (**B**) and habitat preference (**C**) of the extinct taxa of Guadeloupe.

There was a slight but nonsignificant evidence for Brownian motion evolution of log-transformed body size at the species level (*K* = 0.73, *n* = 13, *P* = 0.267). However, after including the data with repetitions within species, the phylogenetic signal for body size was strong (*K* = 2.0, *n* = 61, *P* = 0.001), indicating a strong phylogenetic constraint in the evolution of body size at the level of the whole dataset. Thus, we built two correlation structures: the first one assuming a Brownian motion evolution and the second one assuming evolution under an Ornstein-Uhlenbeck (OU) model. The models incorporating the first correlation structure failed to converge, whereas the models with an OU correlation structure converged and showed evidence for an effect of body size and of habitat on extinction/extirpation status (Fig. 4A). The models fitted by generalized linear model (GLM) and ignoring phylogenetic relationships between species gave very similar results, with body mass and habitat being the two best predictors of extinction/extirpation status (Table 1). The similarity of the results obtained using different approaches shows that although body mass has a phylogenetic component in the species investigated, this does not affect the relationship we identify here. In the generalized linear mixed-effects models (GLMMs), the inclusion of random effects with islands or species (without phylogenetic correlation) did not improve the fit. In these models, there was no evidence of an interaction between body mass and habitat (χ^2^ = 0.8, df = 1, *P* = 0.36). Other variables appeared to be less effective predictors of extinction, but we still observed that islands on which mongoose are present are more affected and that taxa on large or less densely human populated islands tend to be less affected (Table 1). Arboreal and smaller taxa thus clearly appear to have been less affected than terrestrial and large ones. The impact of these two first variables is easy to visualize in the dataset (Fig. 4, B and C).

**Table 1:** Top-ranked models for faunal extinction/extirpation with ΔBIC ≤ 4. Indicated are the df, the log likelihood (LL), the difference in the Bayesian information criterion (BIC) between each model and the highest rank model (ΔBIC), the model weight (*w*BIC), and Nagelkerke’s *R*^2^.

**Model**	**df**	**LL**	**ΔBIC**	***w*BIC**	***R***^**2**^
Habitat + weight	3	−23.832	0	0.44	0.59
Habitat + weight + mongoose	4	−23.347	3.14	0.09	0.60
Habitat + weight + population	4	−23.353	3.15	0.09	0.61
Habitat + weight + island size	4	−23.745	3.94	0.06	0.59

## DISCUSSION

The data from Guadeloupe make it clear that major squamate extinctions followed European colonization of these islands, with at least 50 to 59% of taxa suffering extinction or extirpation in the centuries after 1492. Rates of post-European colonization squamate extinction across the individual islands of the Guadeloupe archipelago are likely many thousands of times higher than background rates, which have been modeled at 0.3 per million years for lineages of reptiles and amphibians in the West Indies (*56*). Lineage persistence times of millions of years through periods of major climate change, including the glacial cycles of the Pleistocene, emphasize the general resilience of taxa on the islands of the Lesser Antilles and the West Indies (*56*) until modern times and highlight the severity of post-European extinction in Guadeloupe. These results demonstrate that failure to consider fossil-derived data, especially regarding poorly studied lineages, probably contributes to a substantial underestimation of the magnitude of the human impact to global biodiversity.

While extinctions in the anthropic era are high, it is important to differentiate pre-Columbian and post-Columbian impacts on squamates in Guadeloupe. No squamate extinctions are recorded in the Indigenous habitation period layers of the sites investigated, and species diversity in our dataset increased in this era due to the introduction of at least two new lizard species (*I. delicatissima* and *T. rapicauda*) to the archipelago. This is in keeping with modern data suggesting that competition with introduced reptiles has little impact on native reptile species compared to other drivers (*57*), but this question remains difficult to investigate in the Caribbean (see Supplementary Text). The pre-Columbian anthropic era data for squamates are, however, in sharp contrast to findings for nonvolant land mammals in the broader insular Caribbean, one of the only oceanic-type island systems to have been colonized by these faunae (*20*). In the Late Quaternary, the West Indies were home to diverse species of endemic eulipotyphlan insectivores, megalonychid sloths, platyrrhine primates, and several families of often large-bodied rodents (*14*, *20*), most of which experienced catastrophic collapse across the Holocene, probably in relation to human colonization and activities (*14*).

Unlike the extinction pattern of Guadeloupe squamates, which is short, sharp, and late, data for land mammals across the Caribbean tend to show a longer-term pattern of ongoing extinction across the Holocene [from c. 5 to 7 thousand years (ka)], albeit one that intensifies with the arrival of agriculturalists and, later, Europeans (*13*, *14*). Caribbean bat extinctions began by 4 ka and were not climate caused (*17*). Cuba saw major extinctions of its land mammals in the past 2 ka, linked to the arrival of Ceramic populations (*13*). Archaeological data demonstrate that Indigenous populations also had a major impact on the marine ecosystems on which they were heavily reliant in the Virgin Islands and the Bahamas (*58*, *59*). While taxonomically uneven data collection remains a problem, there is some evidence to suggest that taxonomic groups were differentially affected by early human populations. Large mammals and bats appear to have been among the first to disappear following human arrival in the Caribbean—although as more accelerator mass spectrometry radiocarbon dates are secured, it becomes clear that this was often a protracted process (*14*)—whereas small-bodied rodents and eulipotyphlan insectivores tended to survive to European times (*14*, *18*).

The data from Guadeloupe, with its unique extended fossil record, allow us to further and more systematically explore extinction dynamics and the biology of resilience to human impacts. The relatively short extinction chronology (<500 B.P.) for the archipelago’s squamates points to a high degree of resilience to the kinds of impacts engendered by Indigenous populations. Pre-Columbian anthropogenic species introductions, which appear to have been so devastating in the colonial period [see also (*15*)], appear not to have had an impact on Guadeloupe squamates nor did pre-Columbian agricultural practices. The historical Guadeloupe dataset is also unique in permitting examination of more fine-grained extinction dynamics, enabling insights into the link between habitat preference, size, and extinction risk through time. This reveals, first, a strong link between squamate habitat preference and human impact, with terrestrial fauna showing greater susceptibility to extinction/extirpation. Our data indicate that arboreal taxa were significantly less susceptible to the environmental impacts of European colonization, with only 17% experiencing extinction/extirpation (when the very small lizards of the genus *Sphaerodactylus* are excluded). Our study also highlights a second factor influencing squamate extinctions/extirpation: weight/size of the species. At least 80% of the medium-sized taxa once present on Guadeloupe are now extinct or extirpated, whereas, in contrast, only 15% of the small taxa present in the Indigenous habitation era are no longer present. Model selection reveals that these two variables explain much of the observed extinction/extirpation pattern, whereas other factors (in particular, the presence of mongoose and the size of the island) appear less significant. This evidence reinforces observations previously made in Guadeloupe and the Caribbean more broadly on the basis of studies of modern lizards (*45*, *60*) and mammals (*14*), linking habitat preference, body size, and extinction risk.

These trends in the data strongly point to a dominant role for recently introduced mammalian predators in Guadeloupe’s post-1492 extinction wave, coincident with findings elsewhere in the Caribbean (*15*). While dogs were present in Guadeloupe from the Saladoid period, along with several other introduced mammals (*38*), European colonial populations introduced several new mammalian predators, including cats and mongooses (*H. auropunctatus*) (*44*), that are known to be excellent squamate hunters, as well as other potential predators such as rats and raccoons (*Procyon lotor*). These predators are mainly terrestrial and are more likely to exploit medium-sized, ground-dwelling taxa that are easier to capture and that provide substantial protein intake. Several other authors have pointed to the important role of the mongoose, introduced originally for pest control, in the recent erosion of Antillean squamate biodiversity (*61*) and broader Caribbean endemic vertebrate biodiversity (*15*). This hypothesis would explain why, despite their large size (*62*), arboreal iguanas are still extant in the Caribbean today.

Introduced predators may also help to explain another trend in the Guadeloupe squamate data. Unexpectedly, the study did not reveal any major influence of island size on the survival of Guadeloupe snakes and lizards, although biogeographic models and fossil data (see Supplementary Text) predict that larger islands should be more resilient and have more areas of refuge from human impacts. In Guadeloupe, however, the least affected islands, Les Saintes and Petite-Terre Islets, are also the smallest. Tellingly, these islands were the least attractive for human settlers, and, critically, both are mongoose free (*44*), while Petite-Terre was never subjected to large-scale agriculture or notable habitat destruction and has been uninhabited since 1972 (*31*), as reflected in the low proportion (20%) of exogenous introduced taxa in its current herpetofauna.

While mongoose presence is important, it is, as highlighted by our models, clearly not the sole driver of squamate extinctions in Guadeloupe. A role for other introduced predators (e.g., cats and rats) should not be overlooked. Furthermore, introduced taxa are strong anthropization benchmarks (*63*) that are correlated with historic and modern land use for intensive agriculture. Impacts of intensive agriculture include habitat fragmentation and destruction, soil quality erosion, and extirpation of the primary insects consumed by terrestrial squamates (*64*). The widespread dry forest that likely hosted some of Guadeloupe’s extinct lizards (*Leiocephalus* and *Pholidoscelis*) is the most sensitive of all the island biomes to soil degradation (*65*) and was strongly affected in the Lesser-Antilles (*66*). These landscape transformations, similar to alien predators, may have had an important impact on biodiversity. Marie-Galante, which is the most heavily affected island in Guadeloupe, with an extinction/extirpation rate of squamate taxa above 70%, differs from the other islands by its early and intensive cultivation since the 17th century. In light of its small size, this likely left few areas of natural refuge. In contrast, the archipelago’s other islands remained mostly undisturbed until the 18th century (*31*).

Our study strongly highlights the need to prioritize study of squamates, as well as other species affected by taxonomic chauvinism (*7*), in both modern and fossil/archaeological contexts. Research on defaunation has highlighted the important loss of large-bodied mammals through body-size downgrading (*30*) and keystone species through trophic downgrading (*67*), but we know much less about size-differential loss and broader ecosystem impacts in reptiles, amphibians, and invertebrates. Increasing evidence nonetheless points to a tendency for larger squamates to fall prey more quickly than smaller ones to extinction processes in a number of island contexts (*60*, *62*), and scientists are beginning to document the impact to ecosystems of such body size–biased species removal. For example, extirpation and extinction of larger-bodied lizards have been shown to affect seed dispersal and plant recruitment in recent studies in the Canary Islands (*68*). Preservation of larger species that ensure effective seed dispersal is crucial to the maintenance of functional seed dispersal services and associated genetic diversity, meaning that they should be an important conservation target (*68*). In Guadeloupe as elsewhere, smaller squamates appear to have been less susceptible to extinction and extirpation, but medium-sized lizards were actually more heavily affected than the largest species. This size-based extinction pattern may reflect the hunting behaviors of introduced mammalian predators, which selected prey size suitable to their own size and needs. Our findings highlight the need to target medium-sized species in conservation efforts in the Guadeloupe Islands.

The relatively late extinctions of snakes and lizards in Guadeloupe also have important broader conservation implications. Our dataset suggests, minimally, several thousand years of human occupation without impact to squamates in Guadeloupe [and as much as almost 5000 years if pollen and charcoal data are correct in indicating earlier human occupation (*34*)]. The persistence of Guadeloupe squamates through major periods of Pleistocene climate oscillation and Indigenous island occupation that, elsewhere in the Caribbean, saw substantial faunal extinctions highlights the resilience of these animals to disturbance. On the other hand, Guadeloupe’s only nonvolant native mammal, the medium-sized rodent, *A. rayi* (*20*), also persisted through to colonial times, only going extinct in the historical era, and marine ecosystems in the Lesser Antilles (*69*), including Guadeloupe (*48*, *70*), also appear to have been relatively resilient to pre-Columbian exploitation. Whatever the nature of pre-Columbian environmental impacts, however, they appear to have been massively ramped up following European colonization, when squamate assemblages on many islands in the archipelago saw marked decline in terms of both taxonomic and morphological diversity (*71*). These declines should cause serious concern. Squamates do not appear to be a sensitive group of fauna in Guadeloupe, and we should accordingly see their disappearance as a warning to humanity about the perils of continued landscape destruction and disturbance that threatens to undermine not only global biodiversity but also our planet’s ability to support human societies.

The fossil record offers an opportunity to understand and learn from past human practices, to examine which human activities offered sustainable lifeways and which were particularly destructive, and to draw on this information to create realistic and data-driven strategies for mediating anthropogenic impacts in a diverse variety of cultural and ecological contexts (*5*, *27*, *28*, *72*). The archaeological record of extinctions from Guadeloupe gives us a clear baseline carrying disturbing news and reason to be deeply concerned, but at the same time, it offers opportunities to explore solutions and to examine the long-term impact of diverse human lifeways. Agriculture per se does not seem to be harmful to Guadeloupe’s squamates, but the shift from diversified agriculture to agricultural specialization and monoculture crops after French colonization in 1635 CE likely negatively affected biodiversity through losses in plant and animal diversity, reduction in pollination services, and changing hydrology, for example (*73*). Perhaps the greatest human impact to squamates, however, derives from the introduction of a variety of “Old World” human commensal species (*15*, *46*). This points to a strong need to prioritize research and conservation measures relating to invasive predators to mitigate global biodiversity loss (*74*).

## MATERIALS AND METHODS

To examine changes to squamate biodiversity over the two last millennia, the squamate osteological remains from 31 Guadeloupe archaeological and paleontological deposits spanning the Late Pleistocene through to modern times were sorted and studied (by C.B. and S.B.) as part of the present study (Fig. 1, table S1, and figs. S1 and S2). Pleistocene and Holocene layers predating the arrival of the first human populations in Guadeloupe around 5000 years ago (*34*) were only available in the paleontological deposits of Marie-Galante Island (Fig. 1, 27 to 29). Pre-Columbian cultural period remains were available for all sites studied. Full details regarding dates, references, and repository locations for each studied deposit are indicated in the Supplementary Materials (table S1). A total of 43,790 bone remains of squamates were isolated and taxonomically identified from the selected assemblages (table S2). These faunal remains had been previously collected from archaeological deposits using 0.5- to 3-mm mesh size sieves and were all retained for further study. Taxonomic identifications were obtained by consulting osteological criteria of taxonomic relevance and by comparison with large sets of modern specimens from the Comparative Anatomy, and Reptiles and Amphibians collections of the Muséum National d’Histoire Naturelle (Paris, France), the University of Bordeaux (Bordeaux, France), the Edgar Clerc Museum (Le Moule, Guadeloupe), the Museum of Comparative Zoology (Harvard, USA), and the Florida Museum of Natural History (Gainesville, USA). Details of these identifications for most taxa were previously discussed in a series of papers (see Supplementary Text for details). The taxonomic attributes discussed in these papers provided the basis for the taxonomic identifications made in the present study. The chronological context of identified bone remains was established on the basis of archaeological data to reconstruct the evolutionary and anthropogenically altered history of Guadeloupe’s squamate diversity during the past millennia. Because of the diversity of archaeological contexts studied and the lack of chronological resolution for most archaeological deposits, we grouped pre-Columbian cultural era finds (combining the Saladoid and Troumassoid periods) into a single chronological bin. Given that we found that most Guadeloupe squamate species went extinct after these periods, this did not significantly affect our study findings. These data were supplemented with published historical and modern data (see Supplementary Text for details).

To better understand the causes of the extinction/extirpation events observed in the fossil archaeological record, we compared alternative models following an approach similar to that previously used to investigate the extinction risks faced by New Zealand lizards (*75*). Briefly, as a response variable, we considered the status of reptile taxa on the different islands defined as extinct (i) or extant (ii) (see table S3). We only included taxa for which past occurrence was clearly demonstrated (see Results) and excluded putative occurrences hypothesized solely on the basis of biogeographic reconstruction. We considered the following explanatory variables: (i) adult body mass of the species in grams (ln transformed), (ii) habitat preferences (terrestrial or arboreal), (iii) activity phase (nocturnal or diurnal), (iv) size of the island in square kilometers (ln transformed), (v) maximum altitude of the island in meters (ln transformed), (vi) whether the island is occupied by mongooses (*76*), and (vii) number of human inhabitants per square kilometer in 1950 (*77*, *78*). We did not consider the body size (length) of the reptile species, as such a variable is not comparable in snake and lizard species and is strongly correlated with their weight. The presence of mongoose on the islands is associated with intensive past and present cultivation of sugar cane, as this animal was originally introduced to eliminate pests from these fields (*76*). We did not consider among the explanatory variables other predators known to strongly affect reptile faunas such as cats and rats since these are present on all Guadeloupe islands. The analysis of the data is challenging because it contains several levels of nestedness and interdependence. To take the phylogenetic relationship between species into account, we used the phylogeny from Pyron *et al*. (*79*) to test for phylogenetic signals. Since we had several observations per species (i.e., the different islands), we added short terminal branches so that the correlation within a species was high. We then built a relevant phylogenetic correlation structure that was used to fit models that consider several variables. Since we considered a non-Gaussian response, we used GLMMs. We opted for the glmmPQL function in the package MASS because it enables the inclusion of a correlation structure [similar to phylogenetic generalized least squares (PGLS)] while being able to model non-Gaussian responses (unlike PGLS). It also enables the inclusion of some predictors as random effects, which is relevant here since the effects of the islands and species variables can be considered random. This framework uses a penalized quasi-likelihood, which does not enable the calculation of information criteria such as Akaike information criterion (AIC) or Bayesian information criterion (BIC). Therefore, we tested the significance of the effects with standard tests on the coefficients. For comparison, we also fitted the same models with a GLM with a binomial response. As there is strong multicollinearity in our dataset, the results obtained should be considered with caution but are still relevant for a comparison with our GLMMs. The R package MuMIN v.1.43.17 executed within the R v. 3.6.3 software environment was used for automated GLMM selection using, as criterion, the BIC adjusted for small sample sizes. All possible combinations of the six explanatory variable retains were considered. Nagelkerke’s *R*^2^ was used as a measure of the explanatory power of the best models.

## SUPPLEMENTARY MATERIALS

Supplementary material for this article is available at http://advances.sciencemag.org/cgi/content/full/7/21/eabg2111/DC1
